# Radiometric Performance Evaluation of FY-4A/AGRI Based on Aqua/MODIS

**DOI:** 10.3390/s21051859

**Published:** 2021-03-07

**Authors:** Bo Zhong, Yingbo Ma, Aixia Yang, Junjun Wu

**Affiliations:** 1 College of Computer Science and Technology, Chongqing University of Posts and Telecommunications, Chongqing 400065, China; zhongbo@radi.ac.cn (B.Z.); macoder95@gmail.com (Y.M.); 2 State Key Laboratory of Remote Sensing Science, Aerospace information Research Institute, Chinese Academy of Sciences, Beijing 100101, China; wujj@aircas.ac.cn

**Keywords:** FY-4A/AGRI, VNIR, DCC, radiometric performance, Aqua/MODIS

## Abstract

Fengyun-4A (FY-4A) is the first satellite of the Chinese second-generation geostationary orbit meteorological satellites (FY-4). The Advanced Geostationary Radiation Imager (AGRI), onboard FY-4A does not load with high-precision calibration facility in visible and near infrared (VNIR) channel. As a consequence, it is necessary to comprehensively evaluate its radiometric performance and quantitatively describe the attenuation while using its VNIR data. In this paper, the radiometric performance at VNIR channels of FY-4A/AGRI is evaluated based on Aqua/MODIS data using the deep convective cloud (DCC) target. In order to reduce the influence of view angle and spectral response difference, the bi-directional reflectance distribution function (BRDF) correction and spectral matching have been performed. The evaluation result shows the radiometric performance of FY-4A/AGRI: (1) is less stable and with obvious fluctuations; (2) has a lower radiation level because of 24.99% lower compared with Aqua/MODIS; 3) has a high attenuation with 9.11% total attenuation over 2 years and 4.0% average annual attenuation rate. After the evaluation, relative radiometric normalization between AGRI and MODIS in VNIR channel is performed and the procedure is proved effective. This paper proposed a more reliable reference for the quantitative applications of FY-4A data.

## 1. Introduction

Fengyun 4A satellite (FY-4A), launched on December 11, 2016, is the first satellite of the Chinese second-generation geostationary orbit meteorological satellite series (FY-4), which is designed to continue the mission of the Fengyun 2 (FY-2), the first generation of geostationary meteorological satellite series. The FY-4A is mainly used (1) to collect multi-spectral and high-precision quantitative observation data of the earth’s surface and clouds; (2) to observe the vertical structure of atmospheric temperature and humidity parameters; (3) to obtain the lightning distribution map through lightning imaging observation; (4) to monitor the space environment and provide observation data for operational space weather forecasting and research, such as broadcast and severe weather warning; (5) to collect various earth environmental parameters automatically. The FY-4A data is useful in various applications on ocean, agriculture, forestry, water conservancy, environment, space science, and so on. The Advanced Geostationary Radiation Imager (AGRI), a multiple channel radiation imager, is one of the primary payloads onboard FY-4A. The FY-4A/AGRI features a precisely designed double-mirror structure, capable of accurate and flexible sensing in two dimensions, and minute-level fast sector scanning. Frequent earth imaging can be performed over 14 bands (including 6 visible/near infrared bands, 2 mid-wave infrared bands, 2 water-vapor bands, and 4 long-wave infrared bands) with off-axis three reflections of primary optic system (http://www.nsmc.org.cn, accessed on 3 November 2020).

Long-term observation of high-quality image data is the premise of quantitative applications of remote sensing [1]. In addition, good radiometric performance is a necessary condition to obtain high-quality data. Like other on-orbit satellite payloads, the radiometric performance of FY-4A/AGRI decays inevitably after launch because of the space environment change and instrument components loss, although pre-launch accurate laboratory calibration is carried out. As a result, regular and reliable on-orbit calibrators are always used to track and evaluate the radiometric performance of on-orbit satellite payloads, like Moderate Resolution Imaging Spectroradiometer (MODIS) onboard Terra and Aqua, Operational Land Imager (OLI) onboard Landsat-8, Multi Spectral Instrument (MSI) onboard Sentinel-2, and so on. For FY-4A/AGRI, on-board black body is available for high frequency calibration in infrared bands. However, there is no onboard calibration system equipped for VNIR bands, vicarious calibration procedure is indispensable, such as site calibration or cross-calibration. The accuracy of the vicarious calibration procedure has great influence on the application of the data. Therefore, a comprehensive evaluation on the FY-4A/AGRI radiometric performance is essential before quantitative applications.

Deep convective clouds (DCC) method is an effective and feasible method to evaluate radiometric capability of sensors. DCC targets are cold and bright targets with high reflectivity, stable reflectivity and good Lambertian characteristics, which are less affected by weather conditions. Abel first attempted to use the deep convective clouds as the calibration reference target [2]. Hu et al. evaluated the radiometric stability of CERES sensors onboard Tropical Rainfall Measuring Mission (TRMM) satellite in short-wave channel using DCC targets after continuous exploration and development [3]. Doelling et al. further analyzed the radiometric characteristics of DCC at different periods and geographical locations (land and sea) in detail, and then calibrated AVHRR data [4,5,6]. In recent years, more and more researchers use DCC targets to evaluate and calibrate the radiometric performance of satellite sensors [7]. In addition, DCC targets can also be used as pseudo-invariant targets to verify the accuracy of satellite radiometric calibration methods [7]. The DCC method has been considered as one of the preferred options by Global Space-based Inter-Calibration Sytem (GSICS) for vicarious calibration in VNIR bands [8]. The accuracy of the DCC method is less than 5% [9].

In this paper, the radiometric performance of FY-4A/AGRI is evaluated from 2018 to 2020 in VNIR band using DCC method. The DCC target is determined by setting the angle range, the infrared brightness temperature threshold of 11um channel and the pixel standard deviation threshold of the visible channel. The BRDF angle correction model (ADM) of CERES thick ice cloud is used to reduce the influence of view angle. Aqua/MODIS data is selected as the reference to describe the FY-4A/AGRI’s radiometric performance attenuation and trend quantitatively. In addition, spectral matching between FY-4A/AGRI and Aqua/MODIS in corresponding band have been done to reduce the influence of spectral response difference.

## 2. Data Set and Method

### 2.1. Data Overview

FY-4Awas successfully launched from the Xichang Satellite launch Center by the Long March 3B improved Type III carrier rocket on 11 December 2016. Then, FY-4A was successfully located over the equator at 99.5°E on 17 December 2016. As a major payload of FY-4A, the AGRI including 6 VNIR bands, 2 short wave infrared (SWIR) bands, 2 medium wave infrared (MIR) bands, two water vapor (WV) bands, and four long wave infrared (LWIR) bands. The settings of VNIR channels are shown in Table 1. In this paper, VNIR-2 (0.55–0.75um) channel is selected to be evaluated.

In this paper, Aqua/MODIS is used as reference data to evaluate FY-4A/AGRI. MODIS onboard Terra and Aqua satellites have been in orbit for 20 years, providing long-term data for global change monitoring. High-precision calibrators onboard MODIS, such as solar diffuse reflector and diffuser stability monitors and spectral radiometric calibrators, have been used to track the spectral response drift and in-orbit bandwidth changes in VNIR bands [10,11]. Moreover, monthly observations have also been performed to improve its calibration accuracy [12]. As a consequence, the calibration accuracy error of the MODIS sensor is maintained at about 2%, which is often used to evaluate the radiometric performance of other sensors and to cross-calibrate other sensors as a reference sensor [13,14,15,16]. In addition, Aqua/MODIS has proved to be more stable than Terra/MODIS [17], so Aqua/MODIS data is selected in this paper.

### 2.2. DCC Target Extraction

The DCC target could be used to evaluate and calibrate radiometric performance for the following reasons: (1) DCC target is bright, which distributes mainly near the equator and moves seasonally with the sun, so it can reflect sunlight well; (2) the influence of water vapor and aerosol on DCC target is relatively small because it locates at the top of troposphere; (3) the DCC target has a high signal-to-noise ratio in the visible band and is isotropic under oblique observation [5]. As the coldest target above the equator, DCC can be easily and well identified by thresholding based on at infrared brightness temperature.

**Figure 1:**
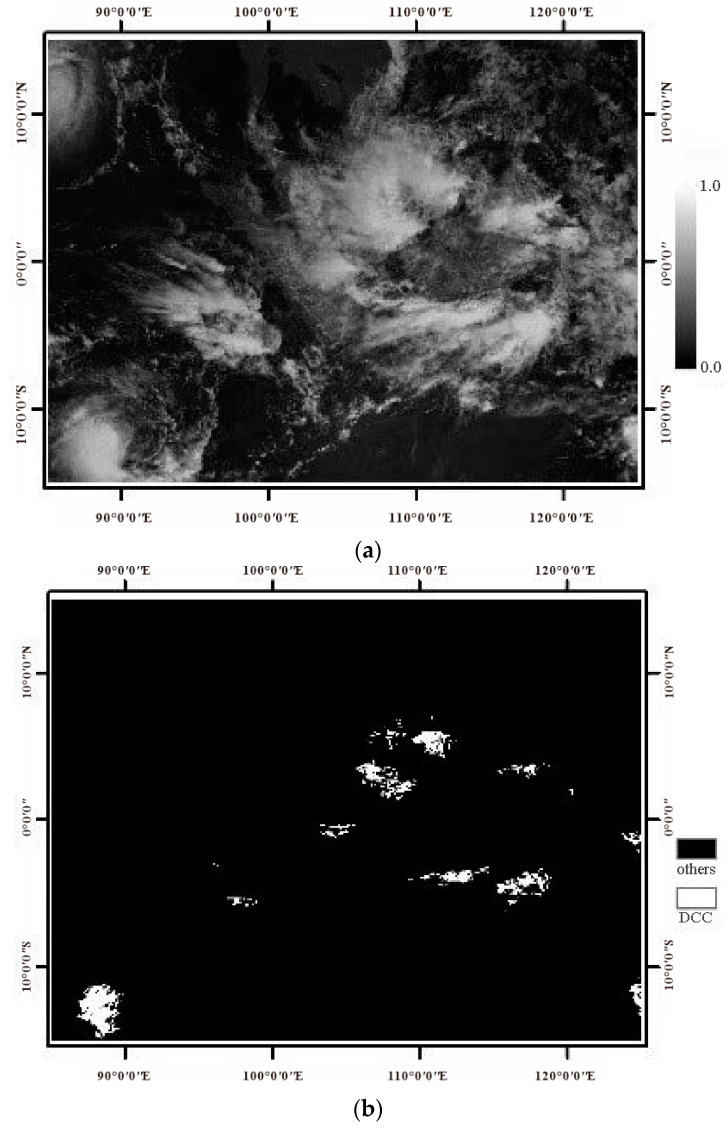
An example of DCC target extraction. (**a**) FY4A visible band image. (**b**) DCC target extraction result.

In this research, the infrared band 12 (10.3~11.3um) of FY-4A/AGRI and band 31 (10.78~11.28um) of MODIS are selected to extract DCC targets (the brightness temperature of this band is less than 210K). In addition, the following rules are set: (1) the longitude is limited to ±20° of the satellite’s operating position (For example, FY-4A/AGRI is located at 99.5°E above the equator, then the location of DCC target is controlled between 80°E and 120°E); (2) the observed zenith angles (VZs) are limited to 40° in order to weaken the influence caused by large viewing angles; (3) the standard deviation of 3*3 pixels in VNIR channel is less than 3% and the standard deviation of brightness temperature in infrared channel is less than 1 K, which is used to avoid the influence of thin clouds and cloud edges, improve the accuracy of DCC target recognition, and eliminate the interference of noise. The DCC selection processes of EOS/MODIS and FY-4A/AGRI are independent of each other. When selecting DCCs of MODIS, the band 31 and band 1 are used. When selecting DCCs of FY4A, the VNIR-2 channel and infrared band 12 are used. Then, the two DCCs are calculated the daily acreage and compared. Taking one FY-4A/AGRI image received on 24 March 2018 for example, the extraction result of DCC target is shown in Figure 1.

### 2.3. BRDF Correction

Although the viewing angle is limited to less than 40° and the DCC target has good Lambertian characteristics, the influence of angular sample still exits. In order to further reduce the viewing angular effect, a BRDF model is used, which is called the angular distribution model (ADM) [18], and was developed for Terra and Aqua using all available CERES rotating azimuth plane radiance measurements [19]. The bidirectional reflection factor in each pixel can be calculated by ADM, and could be used to normalize the reflectance to a single solar zenith angle. The ADM converts the observed radiance into the radiation flux of (TOA) by anisotropy factor, and the expression is defined as follows:

$$F\left(\theta_{o}\right)=\frac{\pi I\left(\theta_{o},\theta ,\phi \right)}{R\left(\theta_{o},\theta ,\phi \right)}$$

where F is the TOA flux emitted or scattered by the Earth-atmosphere per unit area, I is the radiance, $\theta_{o}$ is the solar zenith angle, θ is the view zenith angle, ϕ is the relative azimuth angle defining the view azimuth angle relative to the solar plane, and R is the angular distribution model. The complete CERES Terra/Aqua ADMs are downloaded from the website (https://ceres.larc.nasa.gov/data/angular-distribution-models/, accessed on 21 November 2020). It is a free BRDF calibration parameter LUT table. Given a group of solar zenith angle, view zenith angle and relative Azimuth Angle, the corresponding BRDF correction factor could be found in the LUT table. Then the corrected reflectance could be calculated using the formula:

$$Ref_{cor}=\frac{Ref\left(\theta_{o},\theta ,\phi \right)}{f_{BRDF}\left(\theta_{o},\theta ,\phi \right)}$$

where $f_{BRDF}\left(\theta_{o},\theta ,\phi \right)$ is the BRDF correction factor, $Ref\left(\theta_{o},\theta ,\phi \right)\;\mathrm{and}\;Ref_{cor}$ are the reflectance before and after BRDF correction, respectively. More detailed figures and tabulations of CERES Terra/Aqua ADMs also can be found there.

### 2.4. Spectral Matching

**Figure 2:**
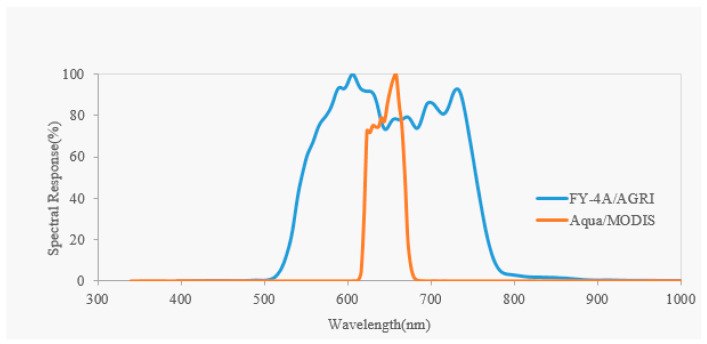
Comparison of spectral response between FY-4A/AGRI and Aqua/MODIS in VNIR channel.

Even small difference between the two sensors will affect the evaluation result. The spectral response function (SRF) between the FY-4A/AGRI (VNIR-2 channel) and MODIS (red channel) (Figure 2). As a consequence, spectral matching must be done to reduce the influence using spectral band adjust factor (SBAF).

**Figure 3:**
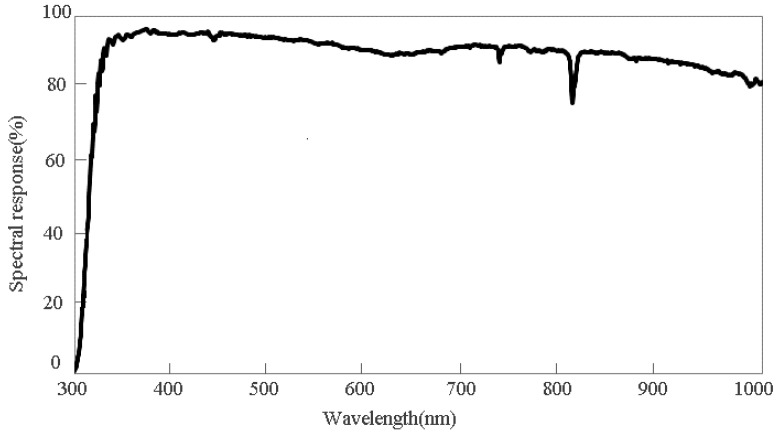
Spectral reflectivity curve of a DCC target provided by David R. Doelling and Rajendra Bhatt (National Aeronautics and Space Administration, NASA) [5].

The SBAF between FY-4A/AGRI and Aqua/MODIS can be calculated according to the following formula:

$$\mathrm{SBAF}=\frac{\int_{\lambda_{1}}^{\lambda_{2}}\rho_{\lambda }*f_{AGRI}\left(\lambda \right)d\lambda /\int_{\lambda_{1}}^{\lambda_{2}}f_{AGRI}\left(\lambda \right)d\lambda }{\int_{\lambda_{3}}^{\lambda_{4}}\rho_{\lambda }*f_{modis}\left(\lambda \right)d\lambda /\int_{\lambda_{3}}^{\lambda_{4}}f_{modis}\left(\lambda \right)d\lambda }$$

where λ is the wavelength,$\;\lambda_{1}$~$\lambda_{2}$ is the spectral range of FY-4A/AGRI, $\lambda_{3}$~$\lambda_{4}$ is the spectral range of Aqua/MODIS, $f_{AGRI}\left(\lambda \right)$ and $f_{MODIS}\left(\lambda \right)$ is the spectral response functions of FY-4A/AGRI and Aqua/MODIS, respectively, $\rho_{\lambda }$ is the normalized spectral response of DCC targets (Figure 3).

The TOA reflectance of FY-4A/AGRI $\rho_{FY4A}$ can be obtained using the formula $\rho_{FY4A}=SBAF*\rho_{MODIS}$, where $\rho_{MODIS}$ represents the TOA reflectance of Aqua/MODIS in red band.

### 2.5. Evaluation Index

The long time series statistics of DCC in three years from 2018 to 2020 were calculated in order to better evaluate the radiometric performance of FY-4A/AGRI. In this research, 3 types of indicators are used [7]:

(1)${\mathrm{Relative}}_{bias}$. ${\mathrm{Relative}}_{bias}$ refers to the deviation degree of the TOA reflectance of FY-4A/AGRI to the 3-year average reflectance of Aqua/MODIS. The relative deviation of FY-4A/AGRI can be calculated by the following formula:

$${\mathrm{Relative}}_{bias}=\frac{f_{FY4A}\left(m_{n}\right)-M_{modis}}{M_{modis}}*100\%$$

where $f_{FY4A}$ is the fitting line of the daily TOA reflectance of FY-4A/AGRI, $M_{modis}$ is the average TOA reflectance of Aqua/MODIS, and $m_{n}$ is the last day in time series of evaluation.

(2) $D_{\mathrm{all}}$(Total attenuation rate) and $D_{\mathrm{year}}$ (Average annual attenuation rate). The total attenuation rate shows the overall attenuation degree of radiometric performance over a period of time, and its calculation formula is as follows:

$$D_{\mathrm{all}}=\frac{f\left(m_{1}\right)-f\left(m_{n}\right)}{f\left(m_{1}\right)}*100\%$$

where f is the fitting line of TOA reflectance, $m_{1}$ is the first day in time series, $m_{n}$ is the last day, and $f\left(m_{1}\right)$ and $f\left(m_{n}\right)$ are the TOA reflectance of the first and last day fitted according to the fitting line, respectively.

$D_{\mathrm{year}}$ means the average annual attenuation, and can be calculate by the following:

$$D_{\mathrm{year}}=(D_{\mathrm{all}}/(m_{n}-m_{1}))*365$$

(3) Stability index (σ): The stability index indicates the degree of dispersion between the scatter and the fitting trend line [7]. The higher the stability index, the greater the deviation is between the real radiometric value and the fitting line, and vice versa. The calculation formula is:

$$\sigma =\sqrt{\frac{1}{n}\overset{n}{\underset{m_{i}=1}{\sum }}{\left(\frac{R\left(m_{i}\right)-f\left(m_{i}\right)}{f\left(m_{1}\right)-\left(R\left(m_{i}\right)-f\left(m_{i}\right)\right)/f\left(m_{1}\right)}\right)}^{2}}$$

where n is the number of days participating in the evaluation, $R\left(m_{i}\right)$ is the average DCC reflectance at day *i*, and $f\left(m_{i}\right)$ is the fitted reflectivity at day *i*.

## 3. Results and Discussion

**Figure 4:**
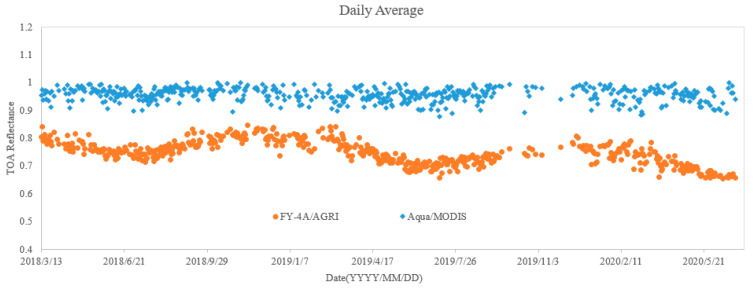
Variation trend of DCCs’ daily TOA reflectance of FY-4A/AGRI and Aqua/MODIS in VNIR-2 channel.

In this study, the daily average DCC TOA reflectance and several evaluation indexes are calculated. The calculated results are shown in Table 2. The average DCC reflectance of 28 months from 2018 to 2020 shown in Figure 4, which reflects the radiometric performance of FY-4A/AGRI in VNIR-2 channel intuitively.

The following conclusions can be drawn from Table 2 and Figure 4:

(1) The attenuation is very small for Aqua/MODIS within 3 years although slight attenuation does exist. As can be seen from Figure 4, the downward trend of the DCC reflectance fitting line of MODIS in 3 years is almost invisible. The total attenuation rate of Aqua/MODIS is only 1.13%, and the annual average attenuation rate is 0.50%. This also shows the radiometric performance of Aqua/MODIS is stable and can be used as reference sensor.
(2) The radiometric performance of FY-4A/AGRI obviously attenuates. As we can see in Table 2, the total attenuation rate of FY-4A/AGRI over a 3-year period is 9.11%, with an annual average attenuation rate of 4.00%. In addition, a significant downward trend can be seen from Figure 4. It is worth noting that the downward trend is not stable, but fluctuating, which may be caused by instability of radiometric performance or change of on-orbit calibration coefficient.
(3) The TOA reflectance of FY-4A/AGRI in DCC targets is much lower than that of Aqua/MODIS. The average value of DCC pixels of Aqua/MODIS is about 0.95, while that of FY-4A/AGRI is about 0.75, −24.99% relative deviation to Aqua/MODIS. This situation is partly due to differences in spectral response between FY-4A/AGRI and Aqua/MODIS. As can be seen from Figure 2, the band range of Aqua/MODIS is between 620 and 670 nm, while the band range of FY-4A/AGRI is between 550 nm and 750 nm, wider than that of Aqua/MODIS. As we all know, the influence of atmosphere on radiation is mainly caused by scattering. When the wavelength is longer, the effect of scattering on radiation decreases, and the effect of atmospheric absorption on radiation increases, resulting in the decrease in TOA reflectance in the VNIR band. The most likely reason that causes the large relative deviation between FY-4A/AGRI and Aqua/MODIS is the calibration coefficient used in VNIR channel. This is happening on other Fengyun series satellites as well. The radiometric performance evaluation results of FY2D, FY2E and FY2F based on MODIS using simultaneous nadir observation (SNO) method also show that the reflectance is much lower than that of MODIS in VNIR band, and this deviation could be reduce though cross radiometric calibration [7].
(4) The TOA reflectance of FY-4A/AGRI fluctuates obviously compared with Aqua/MODIS. Table 2 shows the stability index of FY-4A/AGRI is 0.04, larger than that of MODIS 0.02. As can be seen from Figure 4, the TOA reflectance of Aqua/MODIS fluctuates in a small range near the trend line, while the fluctuation of FY-4A/AGRI is more obvious. This fluctuation has a certain regularity (rising in January, falling in April, rising in July and falling in October). The reason for this phenomenon may be the unstable state of the satellite during the earth shadow period in mid-March–April and mid-September–October every year [20].

**Figure 5:**
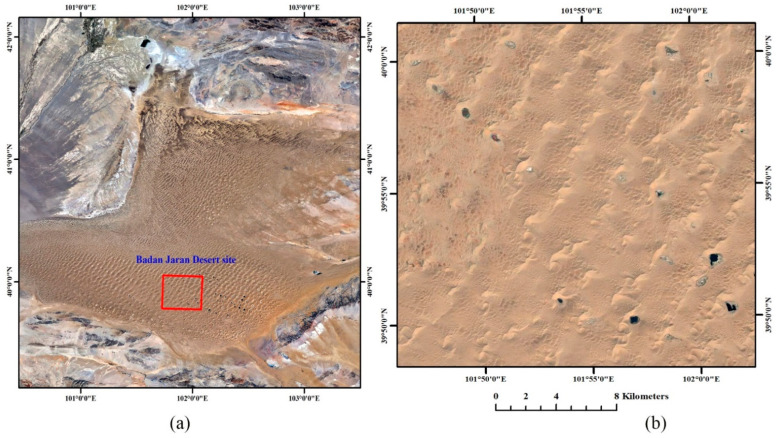
Location (**a**) and close view (**b**) of the Badain Jaran Desert site from Google Earth.

Based on the above evaluation result, radiometric normalization between FY-4A/AGRI and Aqua/MODIS is performed. In order to verify the effectiveness, the TOA reflectance before and after radiometric normalization in time series are compared. The Badain Jaran Desert site (Figure 5) is selected as the test area, which is usually used as test to cross-calibrate and evaluate other sensors [21].

**Figure 6:**
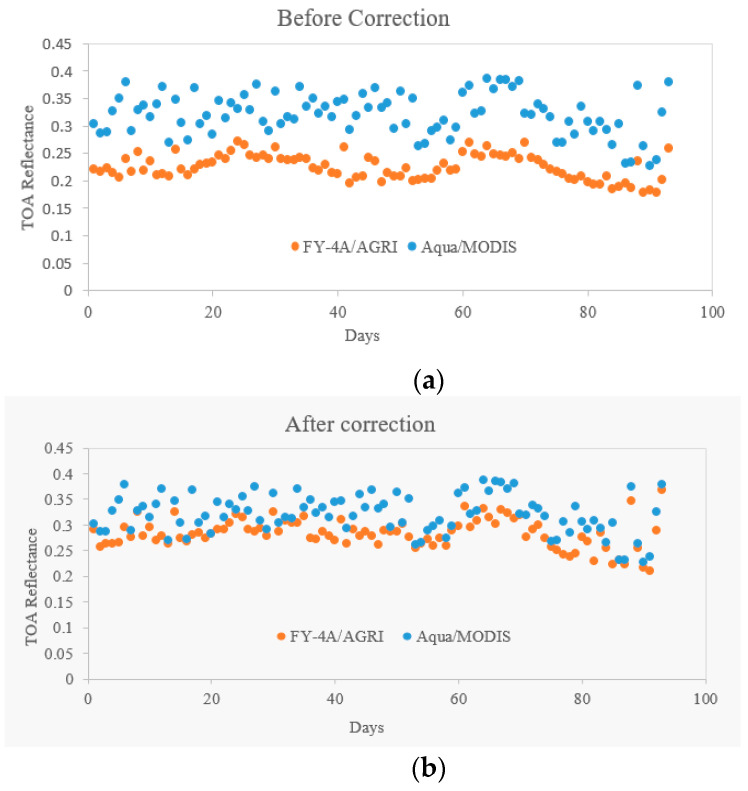
Badain Jaran Desert—Comparison between FY4A and MODIS (**a**) and after correction (**b**).

The TOA reflectance of FY-4A/AGRI before and after radiometric normalization from May 2018 to June 2020 are plotted in Figure 6a,b, respectively. The TOA reflectance of Aqua/MODIS is also plotted as reference.

From Figure 6, the reflectance trend of FY-4A/AGRI is closer with Aqua/MODIS after radiometric normalization than before, and the deviation between FY-4A/AGRI and Aqua/MODIS is smaller. However, From Figure 6b, even after radiometric normalization, the TOA reflectance of FY-4A/AGRI is still lower than that of Aqua/MODIS. This is caused by the difference of view angle. The FY-4A is a geostationary orbit meteorological satellite, whose view angle in Badain Jaran Desert site is always close to 45°. The Aqua is a polar orbit satellite, and MODIS data we selected in this comparison has view angle covering 0~30°. At the same sun angle and weather conditions, the increased radiation indued by the scattering of atmosphere at larger viewing angle is lower than the contribution from relative higher reflected surface (higher than 0.25), so the TOA reflectance becomes smaller while the viewing angle is larger.

The uncertainty in this evaluation procedure mainly comes from: (1) BRDF model, (2) geometric positioning accuracy of Aqua/MODIS and FY-4A/AGRI, (3) spectral matching factor calculation. The uncertainty of BRDF model is less than 4% according to the reference [22]. The spatial resolution of Aqua/MODIS and FY-4A/AGRI are 1km. The DCC selection processes of MODIS and FY-4A are independent of each other. The geometric positioning accuracy just effect the position of DCC pixels, not the reflectance of DCCs. Therefore, we believe this impact is negligible. The uncertainty of spectral matching factor comes from the SCIAMACHY (~1%) according to the reference [5]. Therefore, the overall uncertainty is 4.12%, less than 5% calculated by the formula $\sigma_{O}=\sqrt{\sigma_{1}^{2}+\sigma_{2}^{2}+\sigma_{3}^{2}}$, where $\sigma_{O}$ is overall accuracy, and $\sigma_{1}$,$\sigma_{2}$, and $\sigma_{3}$ are the uncertainty caused by the above three factors. The overall accuracy calculated here is consistent with that in the other literatures [9].

## 4. Conclusions

This paper evaluated the radiometric performance of FY-4A/AGRI in VNIR-2 channel from 2018 to 2020 based on Aqua/MODIS red band. In this process, the BRDF angle correction model (ADM) of CERES Terra/Aqua is used to reduce the influence of view angle, and the spectral matching have been performed in order to reduce the difference of spectral response between FY-4A/AGRI and Aqua/MODIS. Several indicators are calculated, including ${\mathrm{Relative}}_{bias}$, $D_{\mathrm{all}}$(Total attenuation rate), $D_{\mathrm{year}}$(Average annual attenuation rate), and stability index (σ). The evaluation result shows (1) the attenuation is very small for Aqua/MODIS within 3 years (1.13% total attenuation rate and 0.5% average annual attenuation rate), (2) the radiometric performance of FY-4A/AGRI attenuates obviously (9.10% total attenuation rate and 4.0% average annual attenuation rate), (3) the TOA reflectance of FY-4A/AGRI in DCC targets is much lower than that of Aqua/MODIS (−24.99% relative deviation), and (4) the TOA reflectance of FY-4A/AGRI fluctuates obviously compared with Aqua/MODIS. In addition, radiometric normalization between FY-4A/AGRI and Aqua/MODIS is performed and the TOA reflectance in Badain Jaran Desert site before and after radiometric normalization in time series are compared. The comparison shows that the evaluation and normalization is effective, although a small deviation still exists because of the view angle. The overall uncertainty of the method is calculated less than 5%.

The result of radiometric performance evaluation provides a basis for quantitative application of FY-4A/AGRI. In addition, it provides a reference for the cooperative application of FY-4A/AGRI and Aqua/MODIS. Meanwhile, the result could be used to draw on the experience to improve the next generation geostationary orbit meteorological satellites.

## Figures and Tables

**Table 1:** FY-4A/AGRI channel setting.

Channel	Wavelength Range (um)	Spatial Resolution (km)	Main Uses
VNIR-1	0.45–0.49	1	Small particle aerosol, true color synthesis
VNIR-2	0.55–0.75	0.5–1	Vegetation, image navigation and registration, star observation
VNIR-3	0.75–0.90	1	Vegetation, aerosol on water surface

**Table 2:** The statistics of evaluation indexes.

Sensor	Relative Deviation (%)	Total Attenuation Rate (%)	Annual Average Attenuation Rate (%)	Stability Index
AquaMODIS	——	1.13	0.50	0.02
FY-4A/AGRI	−24.99	9.11	4.00	0.04

## Data Availability

Not applicable.
